# Overcoming radioresistance of breast cancer cells with MAP4K4 inhibitors

**DOI:** 10.1038/s41598-024-57000-6

**Published:** 2024-03-28

**Authors:** Yun-Suk Kwon, Min-Gu Lee, Nam-Yi Kim, Gi Suk Nam, Kyung-Soo Nam, Hyunsoo Jang, Soyoung Kim

**Affiliations:** 1 Research Institute of Climate Change and Agriculture, National Institute of Horticultural and Herbal Science, Jeju, Jeju-do 63240 Republic of Korea; 2 Department of Pharmacology, School of Medicine, Dongguk University, Gyeongju, Gyeongsangbuk-do 38066 Republic of Korea; 3 Department of Biomedical Laboratory Science, Honam University, Gwangsan-gu, Gwangju 62399 Republic of Korea; 4 Department of Radiation Oncology, Pohang St. Mary’s Hospital, Pohang, Gyeongsangbuk-do 37661 Republic of Korea

**Keywords:** Radiotherapy, Radioresistance, Breast cancer, MAP4K4, ACSL4, Cancer, Breast cancer, Pharmacology

## Abstract

Mitogen-activated protein kinase kinase kinase kinase 4 (MAP4K4) has recently emerged as a promising therapeutic target in cancer. In this study, we explored the biological function of MAP4K4 in radioresistant breast cancer cells using two MAP4K4 inhibitors, namely PF06260933 and GNE-495. Radioresistant SR and MR cells were established by exposing SK-BR-3 and MCF-7 breast cancer cells to 48–70 Gy of radiation delivered at 4–5 Gy twice a week over 10 months. Surprisingly, although radioresistant cells were derived from two different subtypes of breast cancer cell lines, MAP4K4 was significantly elevated regardless of subtype. Inhibition of MAP4K4 with PF06260933 or GNE-495 selectively targeted radioresistant cells and improved the response to irradiation. Furthermore, MAP4K4 inhibitors induced apoptosis through the accumulation of DNA damage by inhibiting DNA repair systems in radioresistant cells. Notably, Inhibition of MAP4K4 suppressed the expressions of ACSL4, suggesting that MAP4K4 functioned as an upstream effector of ACSL4. This study is the first to report that MAP4K4 plays a crucial role in mediating the radioresistance of breast cancer by acting upstream of ACSL4 to enhance DNA damage response and inhibit apoptosis. We hope that our findings provide a basis for the development of new drugs targeting MAP4K4 to overcome radioresistance.

## Introduction

In modern cancer therapy, radiotherapy is the indispensable treatment modality for achieving loco-regional control and for eradicating residual cancer cells after surgery^1,2^. However, the development of radioresistant cells during radiotherapy has been reported in various cancer types, including non-small cell lung, prostate, breast, and liver cancer, and radioresistance ultimately leads to treatment failure and tumor recurrence^3–7^. Although various factors, such as dysregulations of signaling pathways, the presence of cancer stem cells, an enhanced DNA damage response, and changes in cancer metabolism and microenvironment, have been identified as contributors to the development of radioresistance^8–10^, the precise mechanisms driving radioresistance remain elusive. Given the complexity of radioresistance development, an improved understanding of these mechanisms will facilitate the development of novel therapeutic agents and appropriate countermeasures.

Mitogen activated protein kinase kinase kinase kinase 4 (MAP4K4), also known as hepatocyte progenitor kinase-like/germinal center kinase-like-kinase (HGK), is a serine/threonine protein kinase that belongs to the mammalian sterile 20 protein (STE20p) family of kinases^11^. MAP4K4 was initially identified as a potential therapeutic target in metabolic and cardiovascular diseases because its overexpression is associated with obesity, type 2 diabetes, insulin resistance, thrombosis, and atherosclerosis^12–15^. More recently, MAP4K4 has emerged as a promising therapeutic target in cancer. Following Wright et al.’s initial report on its broad expression in 40 tumor cell lines from a National Cancer Institute human tumor panel^16^, subsequent studies have shown that MAP4K4 is highly overexpressed in various types of cancer, including hepatocellular carcinoma^17^, colorectal cancer^18^, lung cancer^19,20^, prostate cancer^21^, and pancreatic cancer^22^. In addition, high levels of MAP4K4 have also been linked to poorer overall survival and higher tumor recurrence rates among patients with cancer^17,18,20–22^. Several studies have found that MAP4K4 is involved in the proliferation, migration, and invasion of cancer cells, as well as the inhibition of apoptosis^17,23–25^. However, there is no information available on its involvement in resistance to standard cancer therapies. In this study, we discovered that MAP4K4 was highly overexpressed in radioresistant breast cancer cells. Consequently, we investigated the biological function of MAP4K4 in these cells using two MAP4K4 inhibitors, namely PF06260933 and GNE-495.

## Results

### MAP4K4 was overexpressed in radioresistant breast cell lines derived from SK-BR-3 and MCF-7 cells

**Figure 1:**
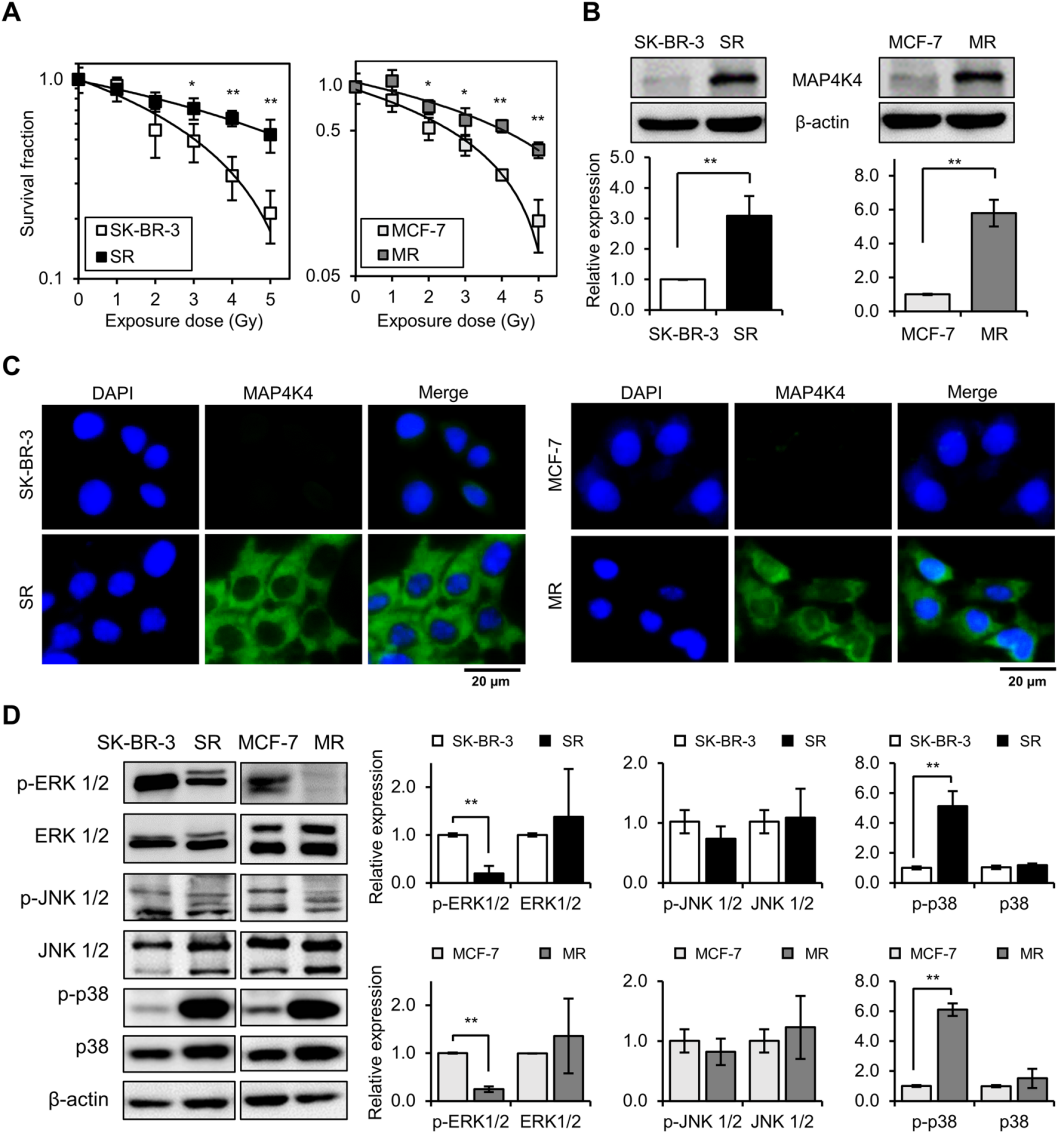
Overexpression of MAP4K4 in radioresistant breast cancer cell lines derived from SK-BR-3 and MCF-7 cells. **(A)** Clonogenic survival assays after irradiating cells with 1, 2, 3, 4, or 5 Gy. Radioresistant SK-BR-3 (SR) cells and radioresistant MCF-7 (MR) cells were resistant to radiation as compared with parental cells (SK-BR-3 and MCF-7). **(B)** Western blot revealed MAP4K4 expression was markedly elevated in SR and MR cells. MAP4K4 expression levels are expressed as fold changes versus parental cells. **(C)** Immunofluorescence analysis of MAP4K4 in parental (SK-BR-3 and MCF-7) and radioresistant (SR and MR) cells**. (D)** Western blot analysis of p-ERK 1/2, ERK 1/2, p-JNK 1/2, JNK 1/2, p-p38, and p38 in SR and MR cells versus parental cells. p-ERK 1/2, ERK 1/2, p-JNK 1/2, JNK 1/2, p-p38, and p38 protein levels are expressed as fold changes versus parental cells. Graphs were plotted using the means ± SDs of three independent experiments. * and ** indicate *P* values of < 0.05 and *P* < 0.01 versus parental cells, respectively.

To establish radioresistant breast cancer cells, an epidermal growth factor receptor 2 (HER2)-positive SK-BR-3 and an estrogen receptor (ER)-positive MCF-7 cells were irradiated using cycles of 4 Gy or 5 Gy, respectively, twice weekly, which resulted in ~ 20% cell survival. Surviving cells were allowed to recover for 6-weeks between cycles and radiosensitivities were monitored. Cycles were continued until meaningful radioresistance had been established. As a result, radioresistant SK-BR-3 (SR) and MCF-7 (MR) cells were successfully established after 6 and 7 cycles, respectively, which resulted in cumulative doses of 48 Gy for SK-BR-3 cells and 70 Gy for MCF-7 cells. When SR and MR cells were irradiated with 1, 2, 3, 4, or 5 Gy and then subjected to clonogenic survival assays, they exhibited significant radioresistance as compared with parental cells (Fig. 1A).

To investigate the role of MAP4K4 in mediating radioresistance, we first compared endogenous MAP4K4 expression levels in parental and radioresistant cells. Interestingly, although SR and MR cells were derived from two different subtypes of breast cancer cell lines, MAP4K4 was similarly dysregulated regardless of subtype. Specifically, the protein expression of MAP4K4 was barely detectable in parental SK-BR-3 or MCF-7 cells but was significantly elevated in SR and MR cells (Fig. 1B). Furthermore, immunofluorescence staining demonstrated that MAP4K4 was mainly localized in cytoplasm in radioresistant cells but was non-detectable in parental cells (Fig. 1C).

As MAP4K4 is known to function through mitogen-activated protein kinase (MAPK) pathways, including MAPK/ERK1/2, MAPK/JNK 1/2, and MAPK/p38, we next checked whether the expressions of MAPKs were elevated in radioresistant cells. Surprisingly, the endogenous expression levels of p-ERK 1/2 and p-JNK 1/2 were found to be lower in SR and MR cells compared to parental cells (Fig. 1D), suggesting that JNK 1/2 and ERK1/2 are not regulated by MAP4K4 in radioresistant cells. However, the expression of p-p38 was significantly increased in SR and MR cells (Fig. 1D).

## MAP4K4 inhibitors selectively targeted radioresistant breast cancer cells

**Figure 2:**
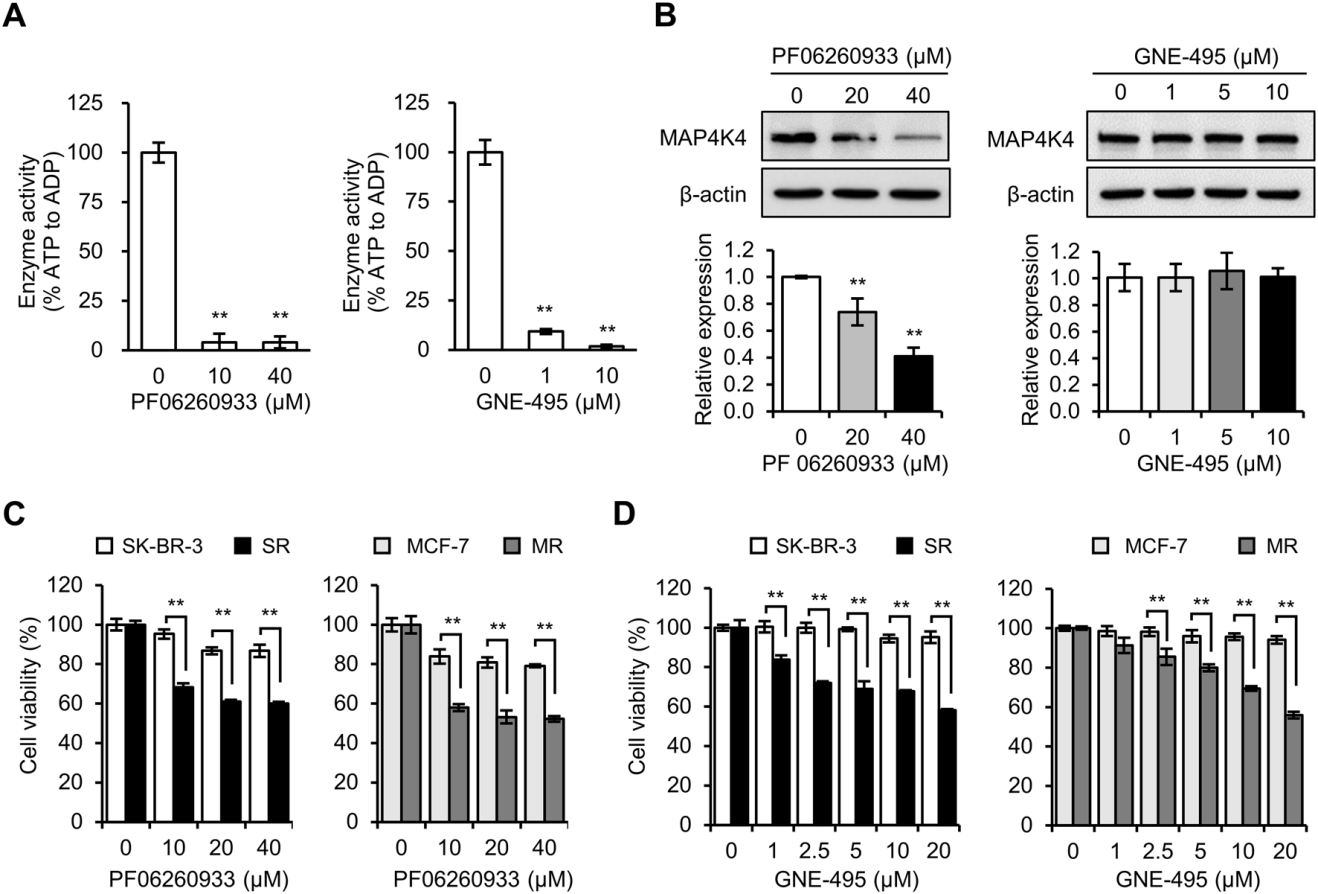
MAP4K4 inhibitors (GNE-495 and PF06260933) selectively targeted radioresistant cells. **(A)** Effects of PF06260933 and GNE-495 on MAP4K4 kinase activities. **(B)** Western blot of MAP4K4 in SR cells treated with PF06260933 or GNE-495. MAP4K4 protein levels are expressed as fold changes versus untreated cells (0 µM). Cytotoxic effects of **(C)** PF06260933 and **(D)** GNE-495 on parental (SK-BR-3 and MCF-7) and radioresistant (SR and MR) cells. Cells were treated with PF06260933 or GNE-495 for 48 h and cell viabilities were assessed using the sulforhodamine B (SRB) assay. Results are presented as means ± SDs, ** indicates P values of  < 0.01 versus untreated cells.

To investigate the role of MAP4K4 in radioresistance in breast cancer cells, we utilized two MAP4K4 inhibitors, namely PF06260933^14^ and GNE-495^26^. Given that MAP4K4 has kinase activity, we initially tested whether these MAP4K4 inhibitors could suppress its kinase activity. As anticipated, both PF06260933 and GNE-495 efficiently inhibited its kinase activity (Fig. 2A). However, to our surprise, western blot analysis revealed an unexpected result: while PF06260933 inhibited MAP4K4 protein expression, GNE-495 did not (Fig. 2B).

To assess the cytotoxic effects of PF06260933 and GNE-495 on SR and MR cells, parental and radioresistant cells were exposed to different concentrations of PF06260933 or GNE-495 for 48 h and then evaluated cell viability. Surprisingly, PF06260933 selectively targeted SR and MR cells, while leaving parental cells mostly unaffected (Fig. 2C). Specifically, at 40 μM concentration, SR and MR cells exhibited cell viabilities around 60% and 50%, respectively, whereas over 80% of parental cells survived (Fig. 2C). Similarly, GNE-495 exhibited greater cytotoxic effects on SR and MR cells compared to parental cells (Fig. 2D), indicating the importance of MAP4K4 kinase activity for the survival of radioresistant cells.

### MAP4K4 inhibitors suppressed tumor growth in mice bearing SR tumors

**Figure 3:**
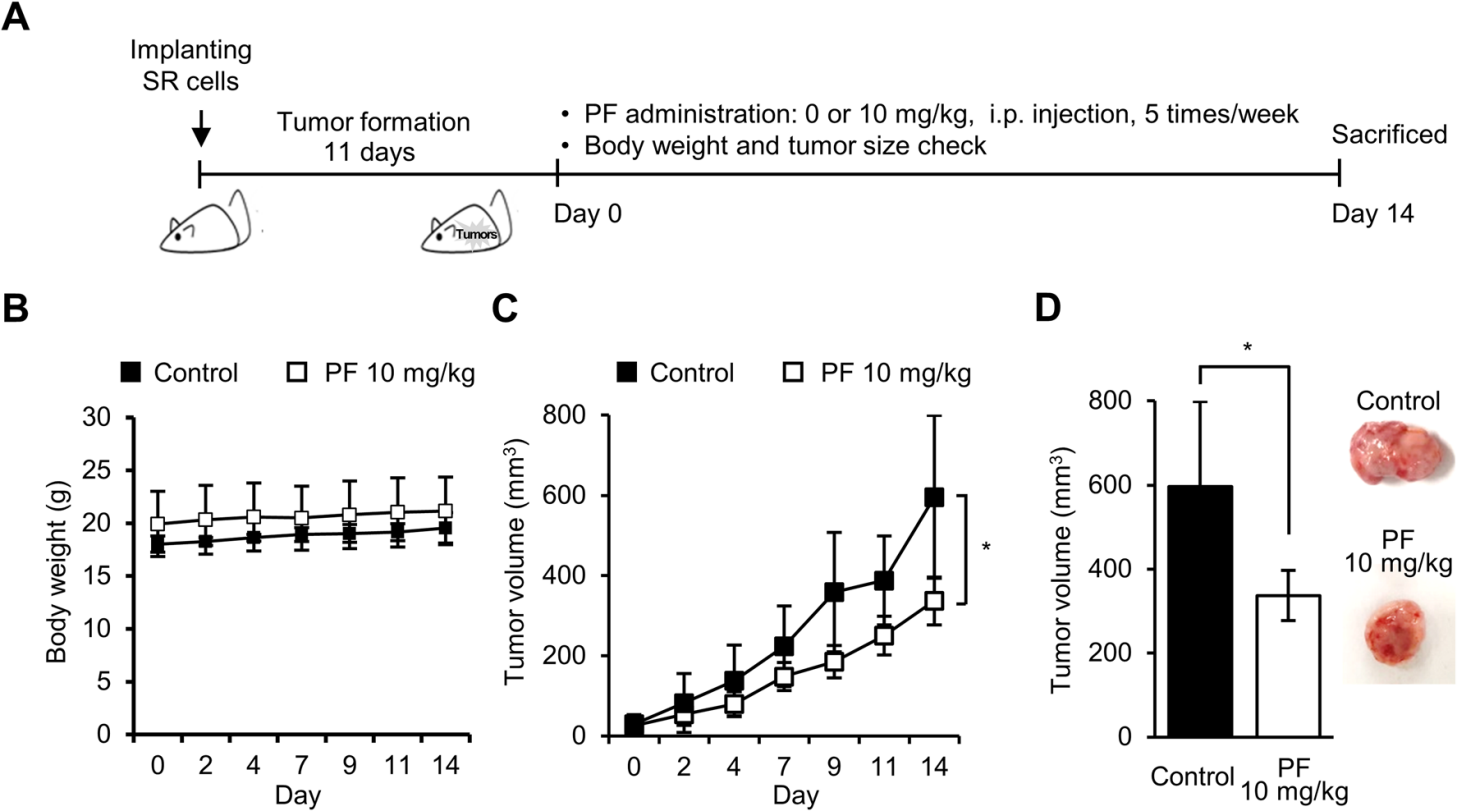
MAP4K4 inhibitor suppressed tumor growth in mice bearing SR tumor. **(A)** The experimental scheme used for testing the anti-tumor effects of PF06260933 (PF) in vivo. **(B)** No loss of body weight occurred in mice treated with PF06260933. **(C)** Tumor growth in SR tumor-bearing *BALB/c* nude mice treated with PF06260933 (n = 4). **(D)** Final tumor volume on day of sacrifice. * indicates *P* value of < 0.05 versus control group.

As MAP4K4 inhibitors showed significant cytotoxic effects on SR and MR cells in vitro, we proceeded to evaluate the potential anti-tumor effects of PF06260933 in vivo. Previously, we reported that SR cells gained tumorigenic potential in vivo, whereas parental SKBR-3 cells were not able to form tumors in mice even when the number of injected cells was increased to 2 × 10^7^/mouse^27^. To assess the in vivo efficacy of PF06260933 on radioresistant breast cancer cells, we implanted SR cells into the mammary gland fat pads of female *BALB/c* nude mice, and when tumors became palpable, we intraperitoneally injected PBS or 10 mg/kg of PF06260933, three times a week for two weeks (Fig. 3A). Notably, the treatment schedules did not affect the body weight of the mice (Fig. 3B). As observed in vitro, PF06260933 treatment in vivo resulted in ~ 40% reduction in tumor growth compared to untreated mice (Fig. 3C and D).

### MAP4K4 inhibitors induced apoptosis by suppressing DNA damage response

**Figure 4:**
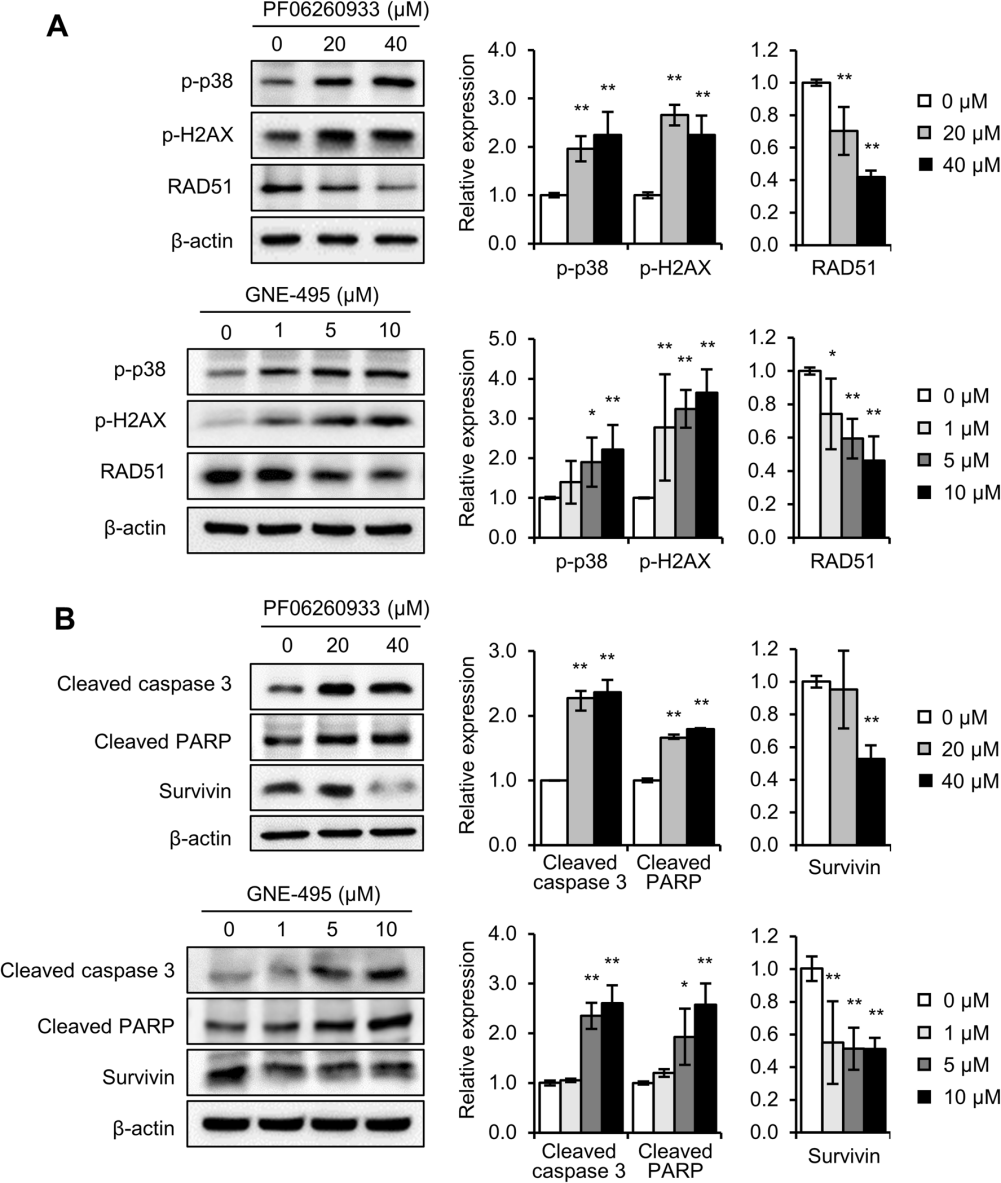
Targeting MAP4K4 suppressed DNA damage response and induced apoptosis in radioresistant cells. **(A)** Expressions of p-p38, p-H2AX, and RAD51 in SR cells treated with PF06260933 or GNE-495 for 24 h. p-p38, p-H2AX, and RAD51 levels are expressed as fold changes versus untreated cells (0 µM). **(B)** Expressions of apoptotic markers, cleaved caspase 3, and cleaved PARP, and survivin in SR cells treated with PF06260933 or GNE-495 for 24 h. Cleaved caspase 3, cleaved PARP, and survivin levels are expressed as fold changes versus untreated cells (0 µM). Results are presented as means ± SDs, and * and ** indicate *p*-values of < 0.05 and < 0.01 versus untreated cells, respectively.

Next, we investigated the mechanisms underlying the cytotoxic effects of MAP4K4 inhibitors on radioresistant cells. In SR and MR cells, only the expression of p-p38 was upregulated (Fig. 1D). Thus, we first checked whether targeting MAP4K4 in radioresistant cells diminished p-p38 expression. Unexpectedly, inhibition of MAP4K4 with PF06260933 or GNE-495 did not inhibit p-p38 expression but instead induced its expression, along with p-H2AX expression (Fig. 4A). Since phosphorylations of H2A.X and p38 increase in response to DNA damage^28,29^, this observation indicated that p-p38 acts as a DNA damage marker rather than a downstream effector of MAP4K4 in radioresistant cells. Furthermore, PF06260933 and GNE-495 suppressed DNA repair response, as indicated by a reduction in RAD51 expression (Fig. 4A). These observations suggest that targeting MAP4K4 with PF06260933 or GNE-495 inhibits the activations of DNA repair systems, resulting in DNA damage accumulation.

As the accumulation of DNA damage often leads to apoptosis, we also explored whether MAP4K4 inhibitors induce apoptosis by evaluating the levels of cleaved caspase 3 and the fragmented form of poly [ADP-ribose] polymerase 1 (PARP), as PARP is cleaved into 89 and 24 kDa fragments by the cleaved form of caspases during apoptosis^30^. Treatment of SR cells with PF06260933 or GNE-495 significantly increased the cleaved form of caspase 3 and the fragmented form of PARP, while the level of the anti-apoptotic protein, survivin, was attenuated (Fig. 4B), suggesting that MAP4K4 regulates apoptosis.

These findings suggest that targeting MAP4K4 accumulates DNA damage by inhibiting the activation of DNA repair systems, and thus, induces the apoptosis of radioresistant cells.

### MAP4K4 functioned as an upstream effector of ACSL4

**Figure 5:**
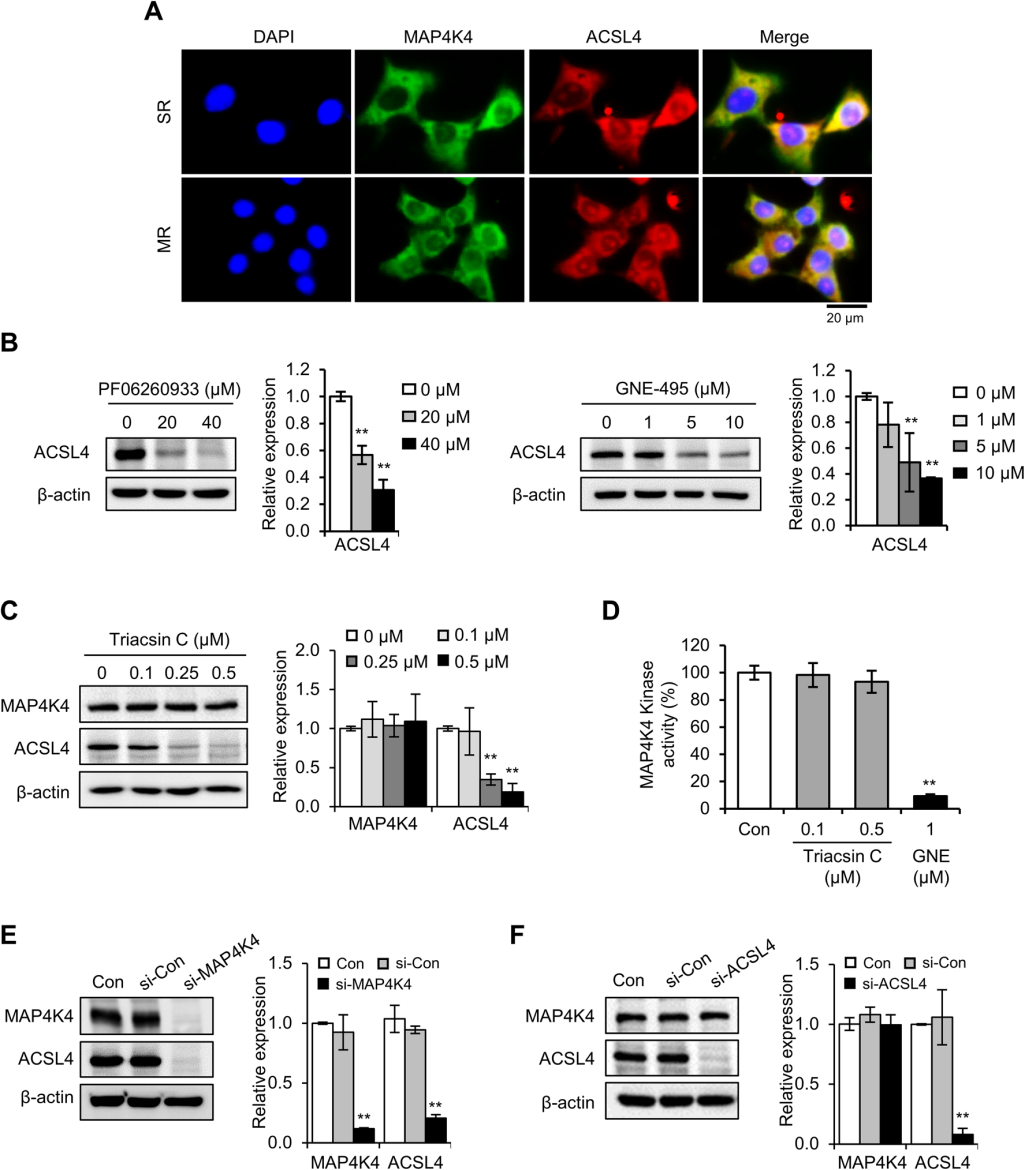
MAP4K4 functioned as an upstream effector of ACSL4. **(A)** Immunofluorescence analysis of MAP4K4 and ACSL4 in SR and MR cells indicated that MAP4K4 co-localized with ACSL4 in radioresistant cells. **(B)** Expressions of ACSL4 in SR cells treated with PF06260933 or GNE-495. **(C)** Expressions of ACSL4 and MAP4K4 in SR cells treated with triacsin C (ACSL4 inhibitor). **(D)** Effect of triacsin C on MAP4K4 kinase activity. The protein expressions of MAP4K4 and ACSL4 following siRNA knockdown. MAP4K4 **(E)** and ACSL4 **(F)** protein levels are expressed as fold changes versus untreated cells (0 µM). Results are presented as means ± SDs, and * and ** indicate *P* values of < 0.05 and < 0.01, respectively.

In our previous study, we demonstrated that acyl-CoA synthetase-4 (ACSL4) contributes to radioresistance in breast cancer cells by enhancing DNA damage response and inhibiting apoptosis^27^. Since MAP4K4 also contributes to radioresistance by regulating DNA damage response and apoptosis, we investigated whether there is an interaction between MAP4K4 and ACSL4 that mediates radioresistance. To explore this, we performed immunofluorescence staining to detect the localization of MAP4K4 and ACSL4 in radioresistant cells. We found that some MAP4K4 staining co-localized with ACSL4 staining in cytoplasm in SR and MR cells, but was undetectable in parental cells (Fig. 5A).

Next, we examined the effects of inhibiting MAP4K4 or ACSL4 on the expression of the other protein. We observed that the inhibition of MAP4K4 with PF06260933 or GNE-495 reduced the expression of ACSL4 (Fig. 5B), while the inhibition of ACSL4 with triacsin C did not affect the expression of MAP4K4, as well as its kinase activity (Fig. 5C and D). These findings suggest that MAP4K4 acts upstream of ACSL4 in radioresistant cells. To confirm this, we performed siRNA transfection targeting MAP4K4 or ACSL4 in SR cells. Consistent with the results from the inhibitors, knockdown of MAP4K4 reduced the expression of ACSL4 (Fig. 5E), while knockdown of ACSL4 did not affect the expression of MAP4K4 (Fig. 5F).

Overall, our results indicate that there is an interaction between MAP4K4 and ACSL4 that mediates radioresistance in breast cancer cells, with MAP4K4 acting as an upstream effector of ACSL4.

### Targeting MAP4K4 overcame the radioresistances of SR and MR cells

**Figure 6:**
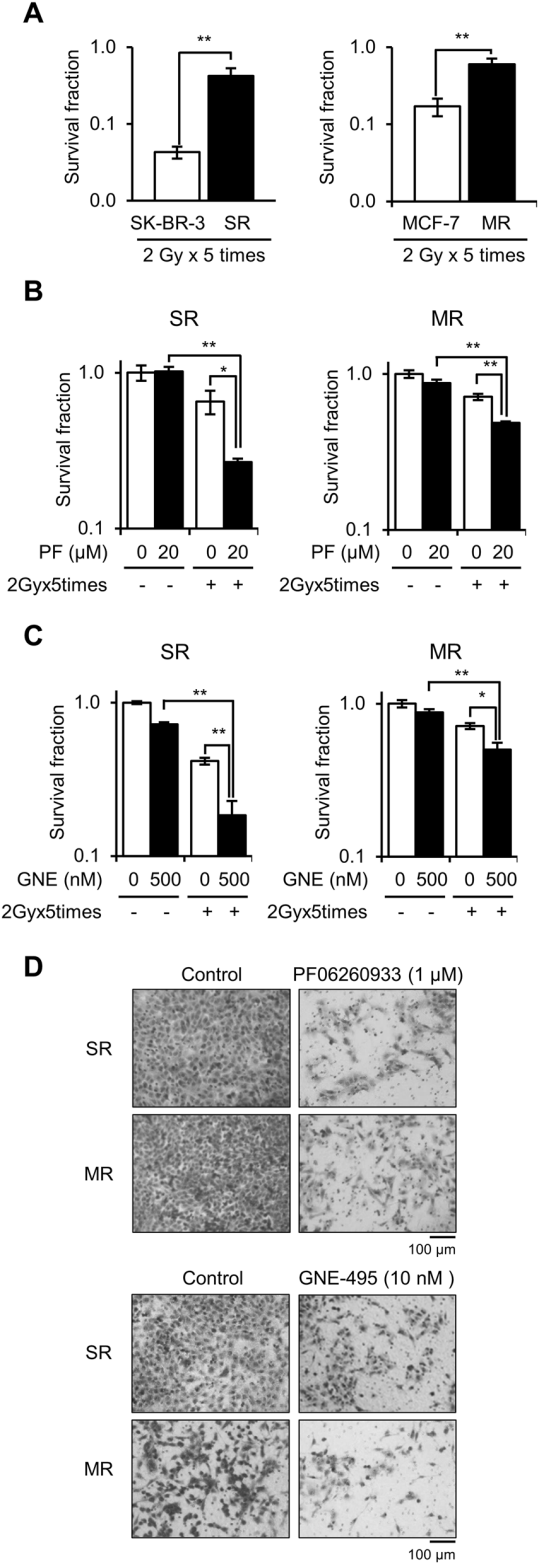
Targeting MAP4K4 overcame the radioresistances of SR and MR cells and suppressed their metastatic properties. **(A)** Radioresistance of SR and MR cells after exposure to 2 Gy/day for 5 days. SR and MR cells were highly resistant to 2 Gy/day × 5 radiation as compared with parental cells. Clonogenic survival assays of SR and MR cells exposed to 2 Gy/day for 5 days in presence or absence of **(B)** PF06260933 (PF) or **(C)** GNE-495 (GNE). Results are presented as means ± SDs, and * and ** indicate *P* values of < 0.05 and < 0.01, respectively. **(D)** Transwell invasion assays of SR and MR cells treated with 1 μM PF06260933 or 10 nM GNE-495 for 24 h.

Encouraged by the observation that MAP4K4 inhibitors selectively targeted radioresistant cells, we investigated whether targeting MAP4K4 could overcome radioresistance in breast cancer. Typically, breast cancer patients receive 1.8–2 Gy of radiation 5 days a week for 5–6 weeks during conventional radiation therapy. Thus, we treated SR, MR, and parental cells with 2 Gy daily for 5 days in the presence or absence of 20 μM PF06260933 or 500 nM GNE-495, and then performed clonogenic survival assays. As expected, SR and MR cells exhibited radioresistance under these conditions (Fig. 6A). About half of the SR and MR cells survived, whereas parental SK-BR-3 and MCF-7 cells were eradicated after exposure (Fig. 6A). However, when MAP4K4 was inhibited, the radioresistance of SR and MR cells were significantly reduced. The survival fractions of SR and MR cells irradiated at 2 Gy × 5 in the presence of PF06260933 were reduced by about 30–60% compared to cells irradiated without PF06260933 (Fig. 6B). Similarly, treatment with GNE-495 also reduced survival fractions of SR and MR cells (Fig. 6C). Additionally, a Transwell invasion assay showed that PF06260933 or GNE-495 efficiently suppressed SR and MR cell migration, while the majority of untreated SR and MR cells easily migrated through membranes (Fig. 6D). Taken together, these observations suggest targeting MAP4K4 can overcome radioresistance and inhibit the metastatic properties of radioresistant breast cancer cells.

## Discussion

**Figure 7:**
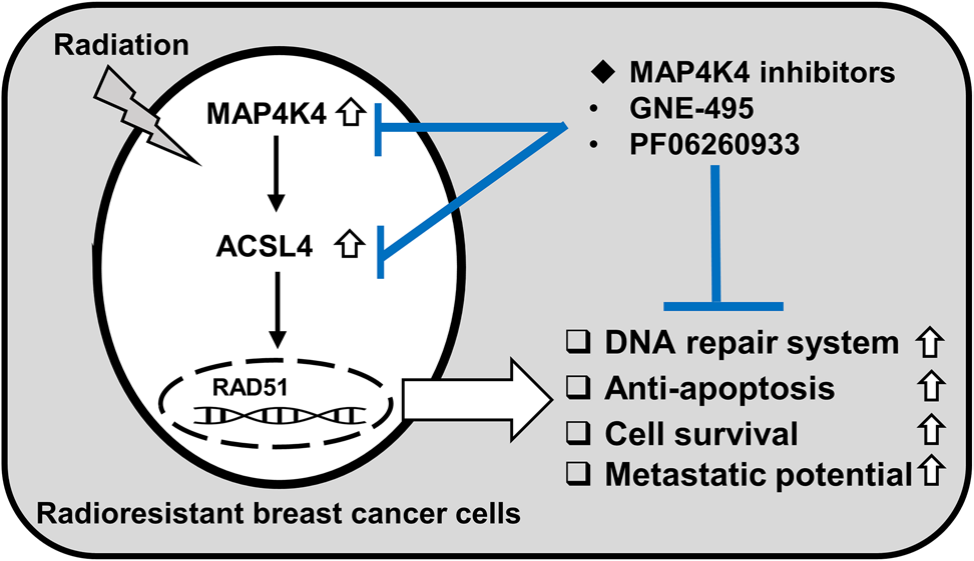
Schematic diagram of the suggested MAP4K4-ACSL4 signaling axis in radioresistant breast cancer cells. We suggest MAP4K4 positively regulates ACSL4 to mediate radioresistance by enhancing DNA damage response and inhibiting apoptosis.

Although MAP4K4 has been identified as a promising target for cancer treatment^31^, its involvement in resistance to conventional cancer therapies remains unclear. This study provides evidence that targeting MAP4K4 can overcome radioresistance of breast cancer by downregulating ACSL4, which suppresses DNA damage response and ultimately induces apoptosis (Fig. 7).

Breast cancers are highly heterogeneous due to variations in the expression of hormone receptors (estrogen and progesterone receptors) and HER2, which necessitates different treatment approaches, such as hormonal therapy, HER2 targeted therapy, or chemotherapy, based on molecular subtypes. Notably, the radioresistant breast cancer cell lines used in this study, SR and MR cells, were derived from two different breast cancer subtypes, HER2-positive SK-BR-3 and ERα-positive MCF-7 cells, respectively. Despite this difference, both radioresistant breast cancer cell lines exhibited a marked upregulation of MAP4K4 expression. Targeting MAP4K4 with two well-known inhibitors, PF06260933^14^ and GNE-495^26^, improved the efficacy of radiotherapy in both radioresistant cancer cell lines. Interestingly, GNE-495 inhibited MAP4K4 kinase activity without affecting its protein expression, whereas PF06260933 inhibited both the kinase activity and protein expression. Nevertheless, both inhibitors overcame radioresistance in SR and MR cells, indicating that MAP4K4 kinase activity is critical for the survival of radioresistant cells.

To better understand the role of MAP4K4 in mediating radioresistance, it is important to identify its downstream mediators. Although it is known that MAP4K4 functions through MAPK pathways, including JNK 1/2, ERK 1/2, and p38, it remains unclear which of the MAPK pathways is activated by MAP4K4 in cancer. The activation of ERK 1/2 and p38 by MAP4K4 has been primarily reported in various biological processes other than cancer^31–34^. Concerning cancer, Gao et al. demonstrated that MAP4K4 activated ERK1/2 in lung adenocarcinoma^19^. In addition, some studies have shown that the activation of JNK 1/2 by MAP4K4 is associated with the motility of cancer cells and epithelial-mesenchymal transition (EMT)^17,23,25^.

In contrast to previous reports, in this study, we found that the levels of p-JNK 1/2 and p-ERK 1/2 were reduced in radioresistant SR and MR cells, while MAP4K4 levels were higher than those in parental cells. These findings indicate that neither JNK 1/2 nor ERK1/2 was affected by MAP4K4 in radioresistant cells. On the other hand, p-p38 expression was upregulated in radioresistant cells, but the inhibition of MAP4K4 with PF06260933 or GNE 495 did not inhibit p-p38 expression. Instead, the p-p38 level was induced along with that of p-H2AX. Since H2A.X and p38 are phosphorylated in response to DNA damage^28,29^, our data suggest that p38 acts as a DNA damage marker rather than a downstream effector of MAP4K4 in SR and MR cells. Taken together, these results suggest that MAP4K4 does not function through MAPK pathways to mediate radioresistance.

Instead of regulating MAPK pathways, we found that there is an interaction between MAP4K4 and ACSL4 that mediates radioresistance in breast cancer cells, with MAP4K4 acting as an upstream activator of ACSL4. Specifically, MAP4K4 inhibition with MAP4K4 inhibitors or siRNA efficiently suppressed the expression of ACSL4 in radioresistant cells, whereas ACSL4 inhibition by ACSL4 siRNA or triacsin C did not alter MAP4K4 expression. ACSL4 is one of five acyl-CoA synthetase long-chain family (ACSL) isoforms that convert long-chain fatty acids into the active form acyl-CoA and catalyze subsequent metabolism^35^. Recent studies have shown that ACSL4 is abnormally expressed in many types of cancer and is related to poor patient survival^36,37^. With regards to breast cancer, studies have reported that ACSL4 overexpression is associated with an aggressive breast cancer phenotype and promotes resistance to hormone therapy^38–40^. In our previous study, we found that ACSL4 enhances DNA damage response and inhibits apoptosis to mediate radioresistance by functioning an upstream effector of Forkhead box protein M1 (FOXM1)^27^. FOXM1 plays an essential role in the regulation of a wide spectrum of biological functions, including cell proliferation, cell cycle progression, cell differentiation, cell survival, and DNA damage repair in cancer cells^41–43^. However, the upstream regulator of ACSL4 remained unidentified. In this study, we identified MAP4K4 as an upstream effector of ACSL4 in radioresistant breast cancer cells. Thus, MAP4K4 contributes to radioresistance in breast cancer by acting upstream of ACSL4 to enhance DNA damage response and inhibit apoptosis.

Overall, our findings suggest that targeting MAP4K4 could be a promising therapeutic strategy for treating radioresistant breast cancer. We hope that this study provides a basis for the development of new drugs that target MAP4K4 to overcome radioresistance.

## Methods

### Materials

Dulbecco's modified eagle's medium (DMEM) and antibiotic–antimycotic solution were purchased from Welgene (Daegu, Korea). Fetal bovine serum (FBS) was obtained from Hyclone Laboratories Inc. (South Logan, UT, USA). PF06260933 (a MAP4K4 inhibitor) was from Axon Medchem (Reston, VA, USA), and GNE-495 (another MAP4K4 inhibitor) and triacsin C (an acyl-CoA synthetase inhibitor) were obtained from Cayman Chemical (Ann Arbor, MI, USA). Sulforhodamine B (SRB) was from Sigma Aldrich (St. Louis, MO, USA). Polyvinylidene fluoride (PVDF) membranes were from Pall Life Sciences (Port Washington, NY, USA). Phosphatase and protease inhibitor cocktails were obtained from GenDEPOT (Barker, TX, USA). Antibodies for phosphorylated (p)-extracellular signal-regulated kinase 1/2 (ERK 1/2), ERK 1/2, p-JNK 1/2, JNK 1/2, p-p38, p38, p-histone family member X (H2A.X), caspase 3, poly (ADP-ribose) polymerase (PARP), and survivin were purchased from Cell Signaling Technology (Beverly, MA, USA). Primary antibodies for acyl-CoA synthetase-4 (ACSL4), RAD51, and β-actin, and siRNA for MAP4K4 (sc-39243), ACSL4 (sc-60619), and control (sc-37007) were purchased from Santa Cruz Biotechnology (Santa Cruz, CA, USA). MAP4K4 antibodies were from Abcam (Cambridge, UK) and Proteintech (Proteintech, IL, USA). HRP-conjugated secondary anti-rabbit and anti-mouse antibodies, Alexa 488-conjugated goat anti-rabbit antibody, Alexa 546-conjugated goat anti-mouse antibody, Prolong Gold anti-fade reagent with DAPI, chamber slides, and BCA protein assay kits were obtained from Thermo Fischer Scientific (Waltham, MA, USA). The ADP-Glo™ kinase assay and active MAP4K4/HGK enzyme were from Promega (Madison, WI, USA) and SignalChem Lifesciences Corporation (Richmond, BC, Canada), respectively. Matrigel and Transwell chambers were from Corning Life Sciences (Bedford, MA, USA).

### Cell culture

SK-BR-3 and MCF-7 human breast cancer cell lines were purchased from the Korean Cell Line Bank (Seoul, Korea) and maintained in DMEM containing 10% fetal bovine serum and 1% antibiotic/antimycotic solution. Media for MCF-7 cells was also supplemented with insulin at 10 μg/mL.

### Establishment of radioresistant cell lines

SK-BR-3 and MCF-7 cells were irradiated using a 21 EX Linac (Varian Medical Systems, Palo Alto, CA, USA) using 6 MV X-rays at 3 Gy/min. During one cycle, cells were irradiated with 4 Gy (SK-BR-3) or 5 Gy (MCF-7) twice a week and allowed to recover for 3 ~ 6 weeks. Radioresistant SK-BR-3 (SR) cells and radioresistant MCF-7 (MR) cells were established by subjecting cells to 6 cycles (cumulative dose 48 Gy) or 7 cycles (cumulative dose 70 Gy), respectively^27^.

### Clonogenic survival assay

To confirm the acquisition of radioresistance, cells were irradiated with 1, 2, 3, 4, or 5 Gy once. To assess the effects of MAP4K4 inhibitors on radioresistance, cells were irradiated with 2 Gy per day for 5 days with or without 20 μM PF06260933 or 500 nM GNE-495. After culture for 10 days, colonies were fixed with 10% formalin and stained with 0.01% crystal violet. A colony was defined as a group of > 50 cells and colonies were counted under a microscope (TS 100, Nikon, Japan). Survival fractions were calculated by comparing the colony numbers of treated and non-treated control cells.

### Chemosensitivity assay

Cells (1000–4000 cells/well) were treated with PF06260933 or GNE-495 for 48 h, fixed with 10% trichloroacetic acid, and stained with SRB for 30 min. After washing with 1% acetic acid to remove excess dye, 10 mM Tris base solution was added to each well to dissolved the protein-bound dye, and absorbances were measured at 510 nm using a microplate reader (Molecular Devices, CA, USA).

### Immunofluorescence

SK-BR-3 and SR cells plated on chamber slides were incubated for 24 h, fixed with ice-cold methanol and acetone for 4 min and 2 min, respectively, blocked with 10% FBS, and incubated with a rabbit MAP4K4 antibody and a mouse ACSL4 antibody at 4 °C overnight. Slides were then washed with PBS and incubated with Alexa 488-conjugated goat anti-rabbit antibody and Alexa 546-conjugated goat anti-mouse antibody for 2 h in the dark. Cells were mounted using Prolong Gold anti-fad reagent with DAPI and photographed under a fluorescence microscope (Zeiss, Germany).

### In vivo testing

Animal experiments were performed using a protocol approved by the Institutional Animal Care and Use Committee of Dongguk University (IACUC no 2018–13). This study complied with local laws and institutional regulations and was conducted following the ARRIVE guidelines (https://arriveguidelines.org). Female *BALB/c* nude mice (7 weeks old) were purchased from Orient Bio Inc. (Sungnam, Korea) and allowed to acclimatize under a 12 h light/dark cycle at 25 ± 2 °C and 50 ± 5% relative humidity for a week. SR cells (2 × 10^6^) suspended in serum-free media were directly injected into #4 mammary fat pads^44^. When tumors were palpable, mice were randomly divided into two groups (n = 4 per group) and intraperitoneally administered 10 mg/kg of PF06260933 in PBS or the same volume of PBS, three times weekly for two weeks. Mice were sacrificed using cervical dislocation after two weeks, and the tumors were excised. Tumor sizes and body weights were measured three times weekly and tumor volumes were calculated using the formula: tumor volume (mm^3^) = shortest length^2^ × the longest length × 0.5.

### Western blot analysis

Parental and radioresistant cells seeded in 60-mm culture dishes were harvested for western blot after overnight growth or treatment for 24 h with PF06260933, GNE-495, or triacsin C. Cells were lysed with RIPA buffer (150 mM NaCl, 1% Triton X-100, 1% sodium deoxycholate, 0.1% SDS, 50 mM Tris–HCl, pH 7.5 and 2 mM EDTA) supplemented with phosphatase and protease inhibitor cocktails. Lysates were centrifuged at 13,000 rpm at 4 °C for 10 min to remove cell debris. Same amounts of proteins were separated by SDS-PAGE and then transferred to PVDF membranes. After blocking with 5% non-fat skim milk, membranes were incubated overnight at 4 °C with the following primary antibodies; MAP4K4, p-ERK 1/2. ERK 1/2, p-JNK 1/2, JNK 1/2, p-p38, p38, ACSL4, p-H2AX, p-p38, caspase 3, poly (ADP-ribose) polymerase (PARP), survivin, RAD51, FOXM1, and β-actin. Blots were washed with 1× TTBS and incubated with HRP-conjugated secondary anti-rabbit or anti-mouse antibody for 1 h at room temperature. Protein bands were developed using a Luminescent Image Analyzer LAS-4000 (Fujifilm, Tokyo, Japan).

### MAP4K4 kinase assay

The activity of MAP4K4 was measured using the ADP-Glo™ kinase assay from Promega^45^. For the kinase reaction, 100 μg/mL of active MAP4K4/HGK enzyme, 1 mg/mL of myelin basic protein (MBP) as substrate, 10 μM of ATP, and inhibitor (PF06260933, GNE-495, or triacsin C) were added to a 96-well plate containing kinase assay buffer (5 mM 3-morpholinopropanesulfonic acid, 2.5 mM β-glycerol phosphate, 5 mM MgCl_2_, 1 mM EGTA, 0.4 mM EDTA, 0.05 mM 1,4-dithiothreitol, 40 μg/mL BSA, pH 7.2). After incubating the plate at room temperature for 30 min, ADP-Glo reagent was added to terminate the kinase reaction and deplete remaining ATP. ADP was then converted to ATP using the kinase detection reagent, which generated light from the ATP using the luciferin/luciferase reaction. This luminescence was proportional to the activity of MAP4K4 and was measured using a multi-detection microplate reader (Molecular Devices).

### Small interfering RNA (siRNA) transfection

RNA interference-mediated gene silencing was performed using ACSL4 siRNA. SR cells were seeded in 60-mm culture dishes, grown overnight, and then transfected with 100 nM ACSL4 siRNA or control siRNA for 72 h. The cells were harvested and subjected to western blot.

### Statistical analysis

The significances of differences were determined using the Student's t-test or one-way ANOVA with the LSD post hoc test using SPSS V18.0 software (SPSS, Inc., Chicago, IL, USA). Results are presented as means ± standard deviations (SDs). *P* values of < 0.05 were considered statistically significant.

### Approval for animal experiments

The in vivo study was approved by Institutional Animal Care and Use Committee (IACUC) of Dongguk university (IACUC no 2018-13). This study was conducted appropriately and in accordance with local laws and institutional requirements.

### Supplementary Information

Supplementary Figures.

## Data Availability

The data supporting the findings of this study will be made available by the corresponding author upon a reasonable request.
