# Supplementary material for: Overcoming radioresistance of breast cancer cells with MAP4K4 inhibitors
Source: Sci Rep. 2024 Mar 28;14:7410. doi: 10.1038/s41598-024-57000-6 (PMC10978830; doi:10.1038/s41598-024-57000-6)

Supplementary data Original blots

Figure 1B

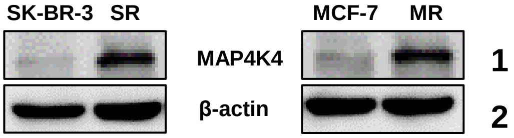

1. MAP4K4 (151 kDa)

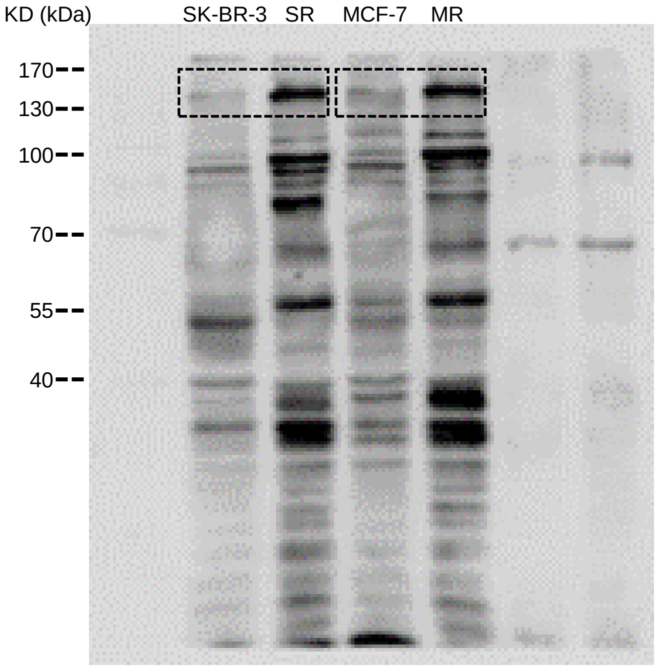

2. β-actin (42 kDa)

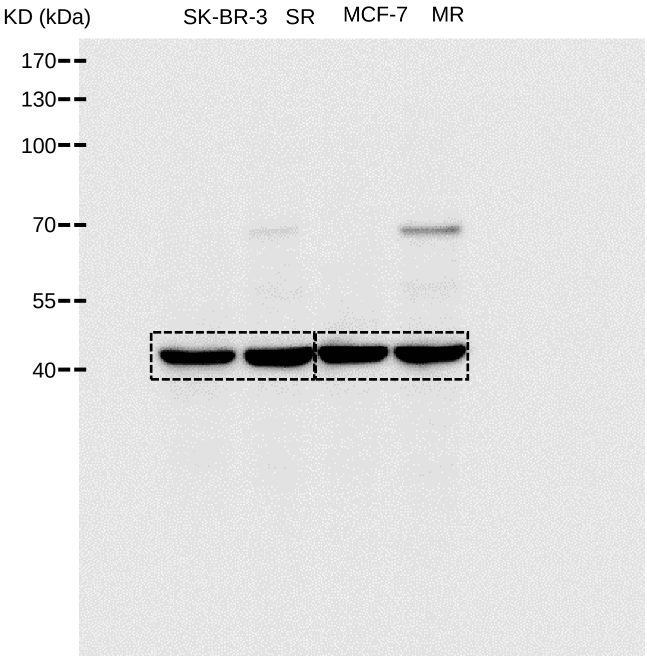

Figure 1D

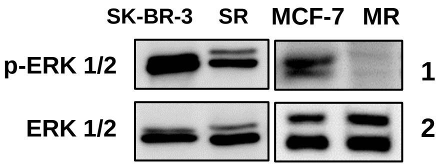

1. p-ERK1/2 (44/42 kDa)

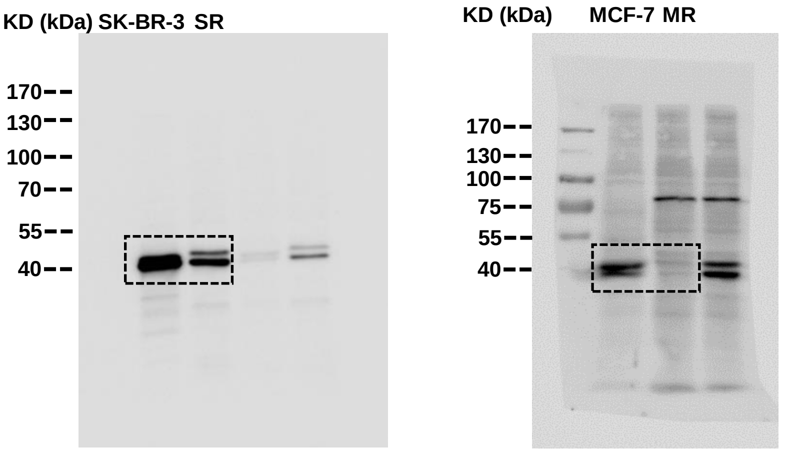

2. ERK1/2 (44/42 kDa)

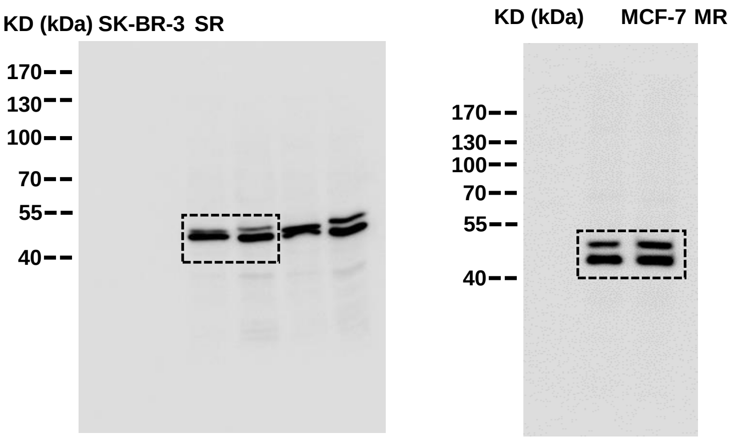

Figure 1D

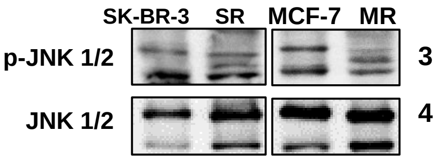

3. p-JNK1/2 (54/46 kDa)

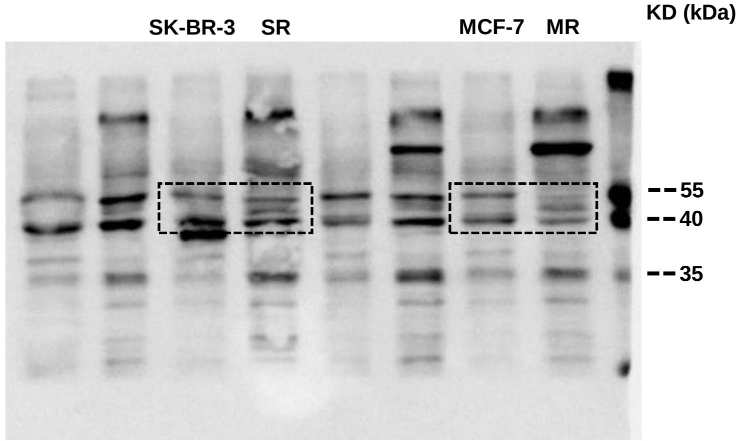

4. JNK1/2 (54/46 kDa)

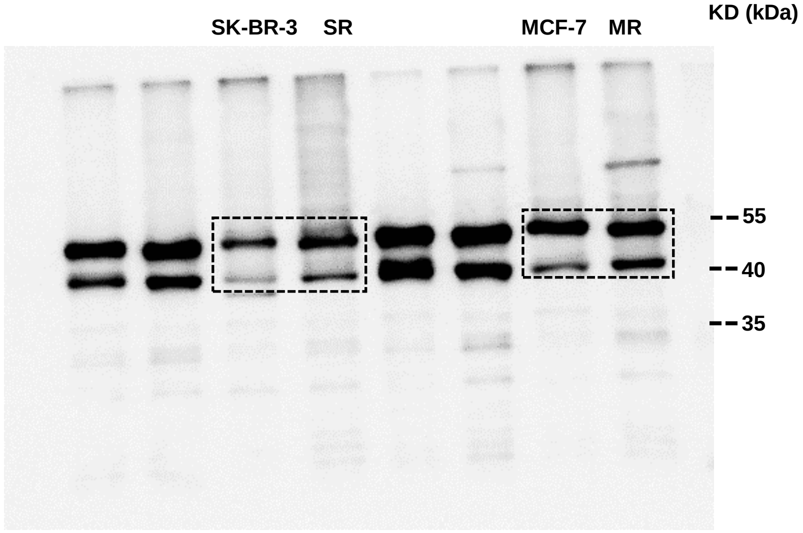

Figure 1D

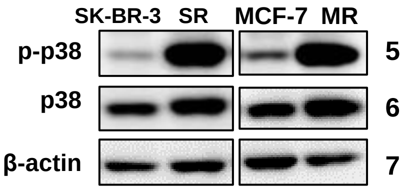

5. p-p38 (43 kDa)

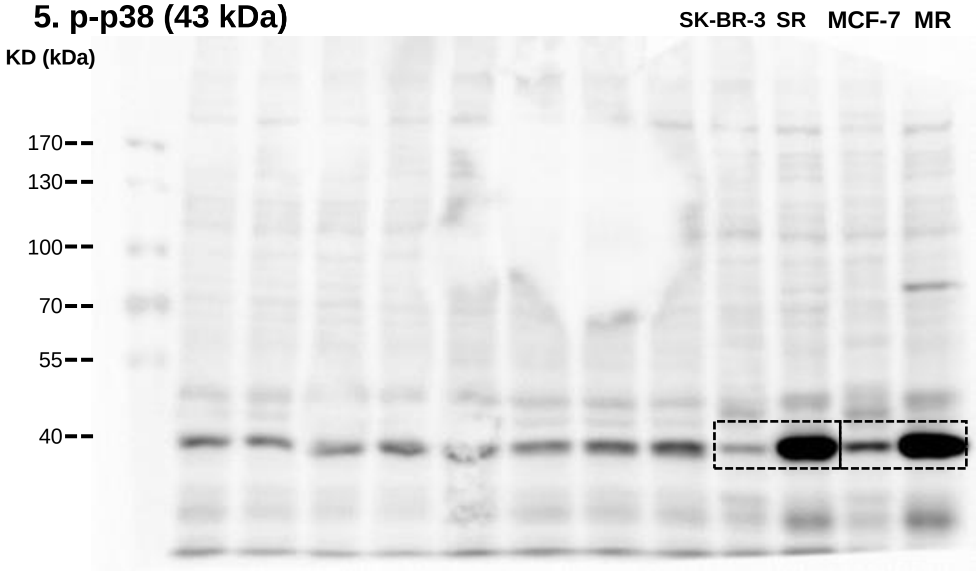

6. p-p38 (43 kDa)

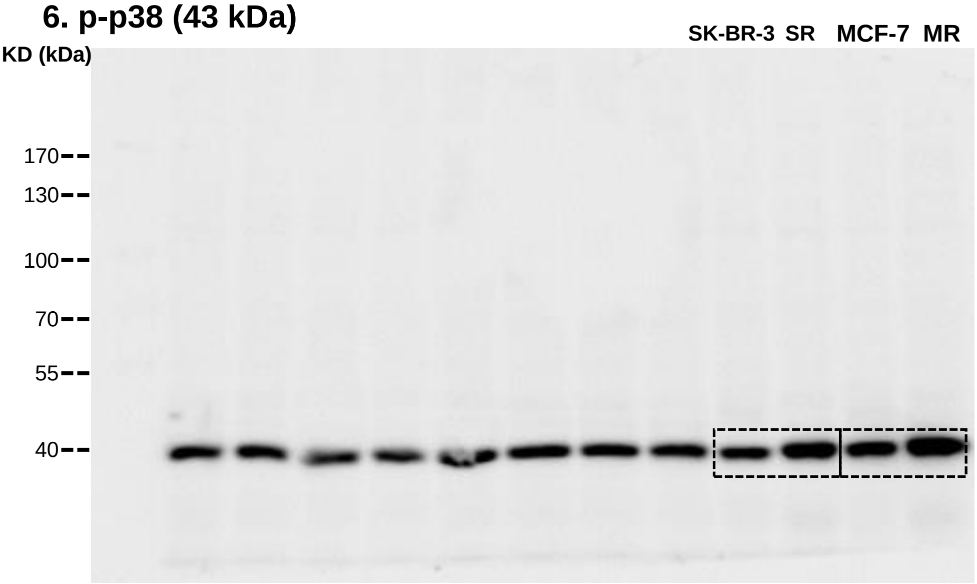

7. β-actin (42 kDa)

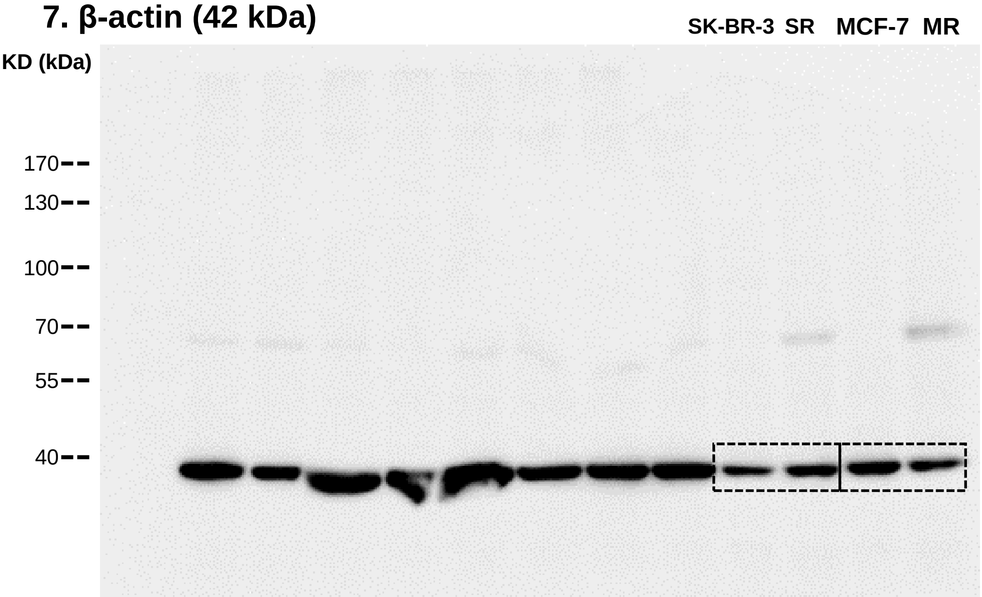

Figure 2B

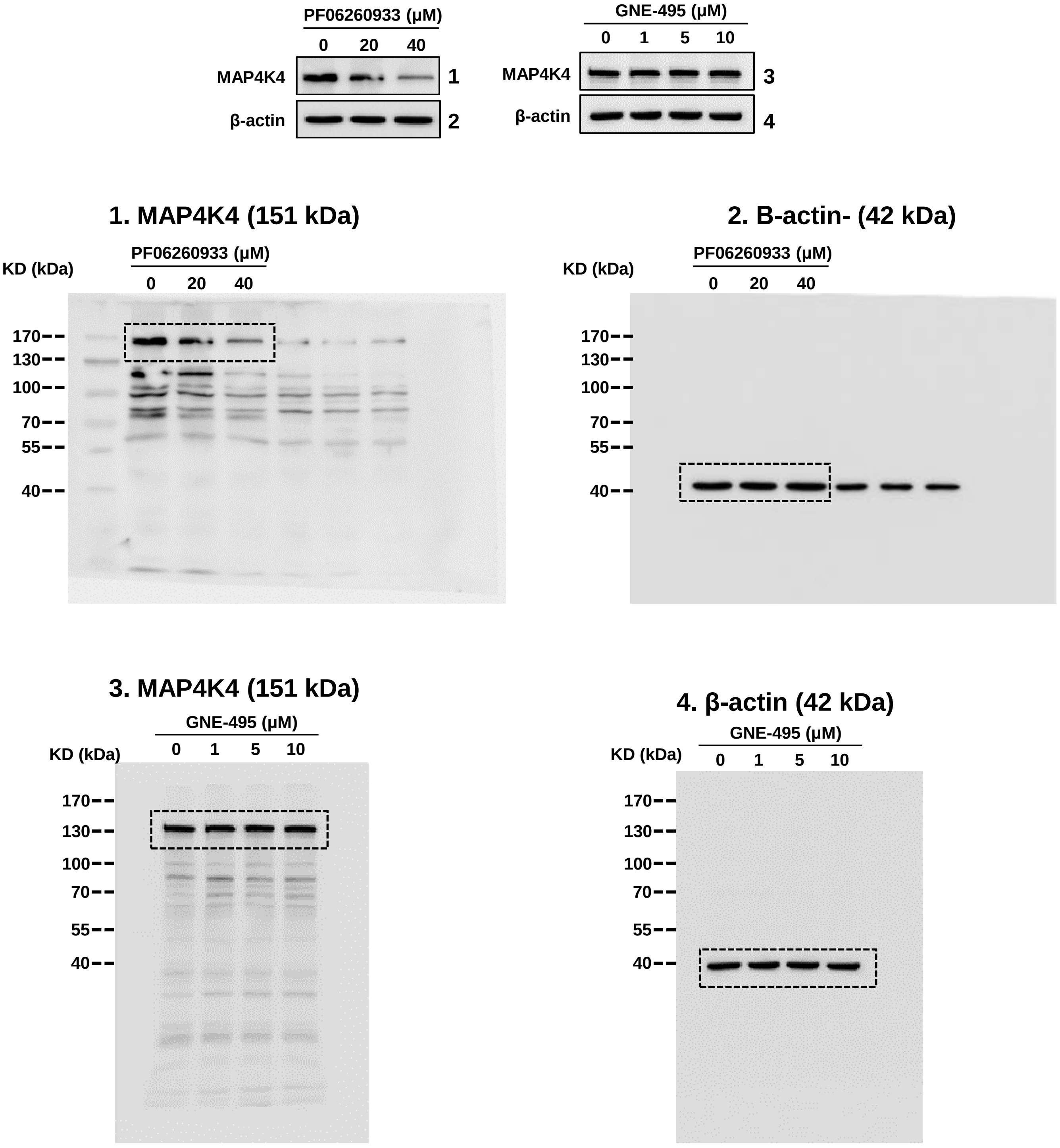

PF06260933 (μM)
0
20
40
MAP4K4
β-actin
1
2
GNE-495 (μM)
0
1
5
10
MAP4K4
β-actin
3
4
1. MAP4K4 (151 kDa)
KD (kDa)
PF06260933 (μM)
0
20
40
170
130
100
70
55
40
2. B-actin- (42 kDa)
KD (kDa)
PF06260933 (μM)
0
20
40
170
130
100
70
55
40
3. MAP4K4 (151 kDa)
GNE-495 (μM)
KD (kDa)
0
1
5
10
170
130
100
70
55
40
4. β-actin (42 kDa)
GNE-495 (μM)
KD (kDa)
0
1
5
10
170
130
100
70
55
40

Figure 4A

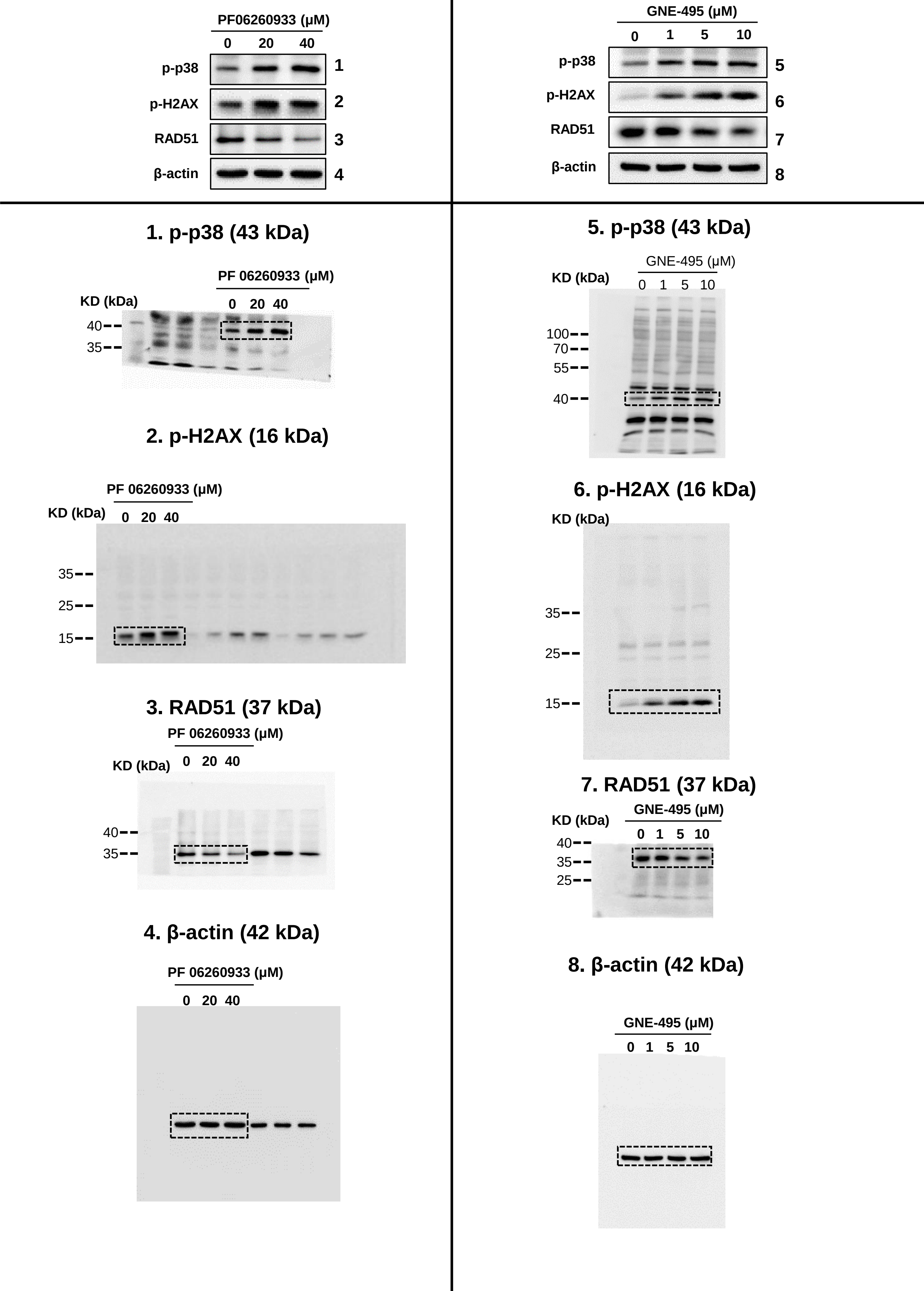
PF06260933 (μM)
0 20 40
p-p38 1
p-H2AX 2
RAD51 3
β-actin 4
GNE-495 (μM)
0 1 5 10
p-p38 5
p-H2AX 6
RAD51 7
β-actin 8
1. p-p38 (43 kDa)
PF 06260933 (μM)
KD (kDa)
0 20 40
40
35
2. p-H2AX (16 kDa)
PF 06260933 (μM)
KD (kDa)
0 20 40
35
25
15
3. RAD51 (37 kDa)
PF 06260933 (μM)
KD (kDa)
0 20 40
40
35
4. β-actin (42 kDa)
PF 06260933 (μM)
0 20 40
5. p-p38 (43 kDa)
GNE-495 (μM)
KD (kDa)
0 1 5 10
100
70
55
40
6. p-H2AX (16 kDa)
KD (kDa)
35
25
15
7. RAD51 (37 kDa)
GNE-495 (μM)
KD (kDa)
0 1 5 10
40
35
25
8. β-actin (42 kDa)
GNE-495 (μM)
0 1 5 10

Figure 4A

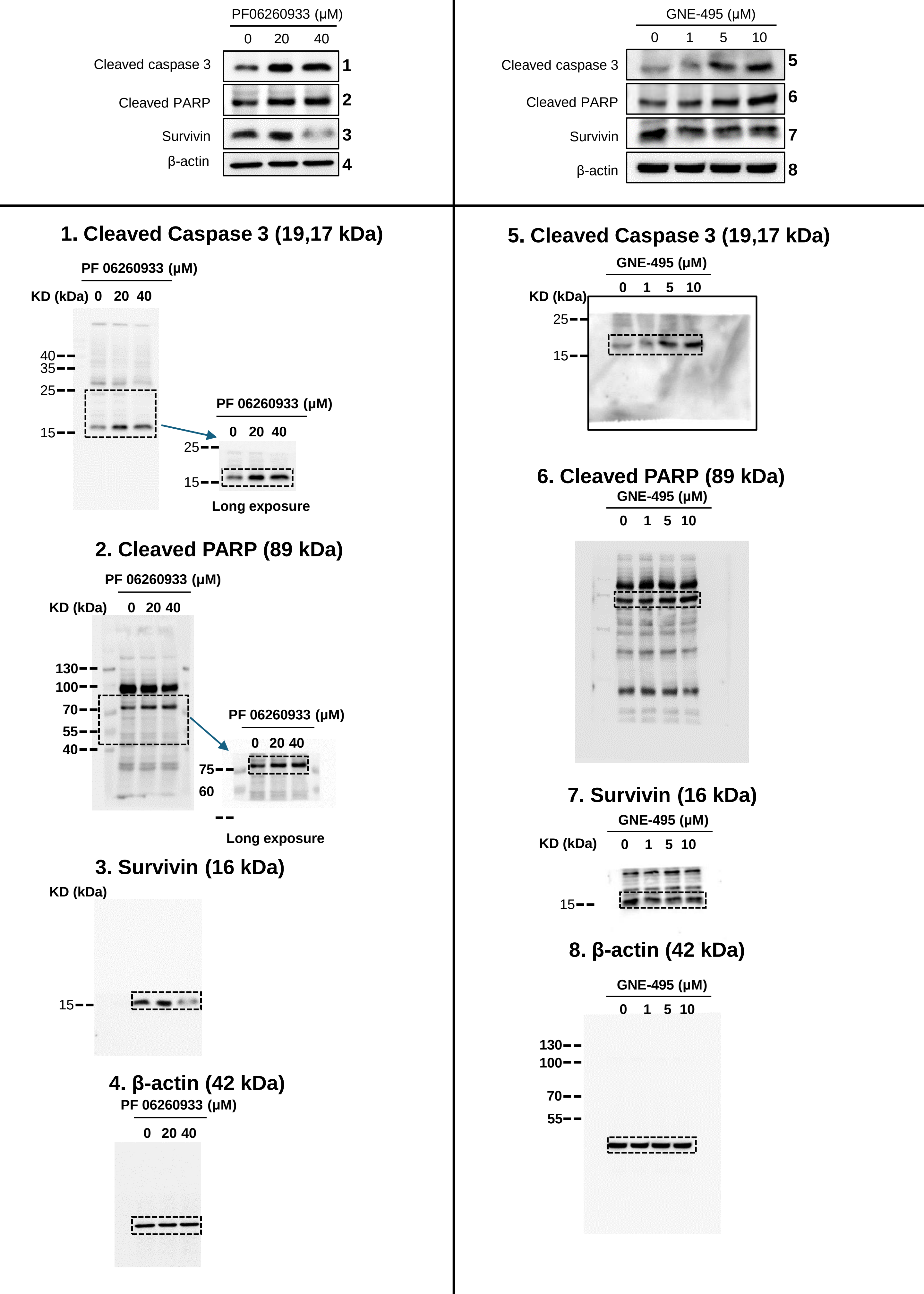

PF06260933 (μM)
0
20
40
Cleaved caspase 3
Cleaved PARP
Survivin
β-actin
1
2
3
4
GNE-495 (μM)
0
1
5
10
Cleaved caspase 3
Cleaved PARP
Survivin
β-actin
5
6
7
8
1. Cleaved Caspase 3 (19,17 kDa)
PF 06260933 (μM)
KD (kDa) 0 20 40
40
35
25
15
PF 06260933 (μM)
0 20 40
25
15
Long exposure
2. Cleaved PARP (89 kDa)
PF 06260933 (μM)
KD (kDa) 0 20 40
130
100
70
55
40
PF 06260933 (μM)
0 20 40
75
60
Long exposure
3. Survivin (16 kDa)
KD (kDa)
15
4. β-actin (42 kDa)
PF 06260933 (μM)
0 20 40
5. Cleaved Caspase 3 (19,17 kDa)
GNE-495 (μM)
0 1 5 10
KD (kDa)
25
15
6. Cleaved PARP (89 kDa)
GNE-495 (μM)
0 1 5 10
7. Survivin (16 kDa)
GNE-495 (μM)
KD (kDa) 0 1 5 10
15
8. β-actin (42 kDa)
GNE-495 (μM)
0 1 5 10
130
100
70
55

Figure 5B

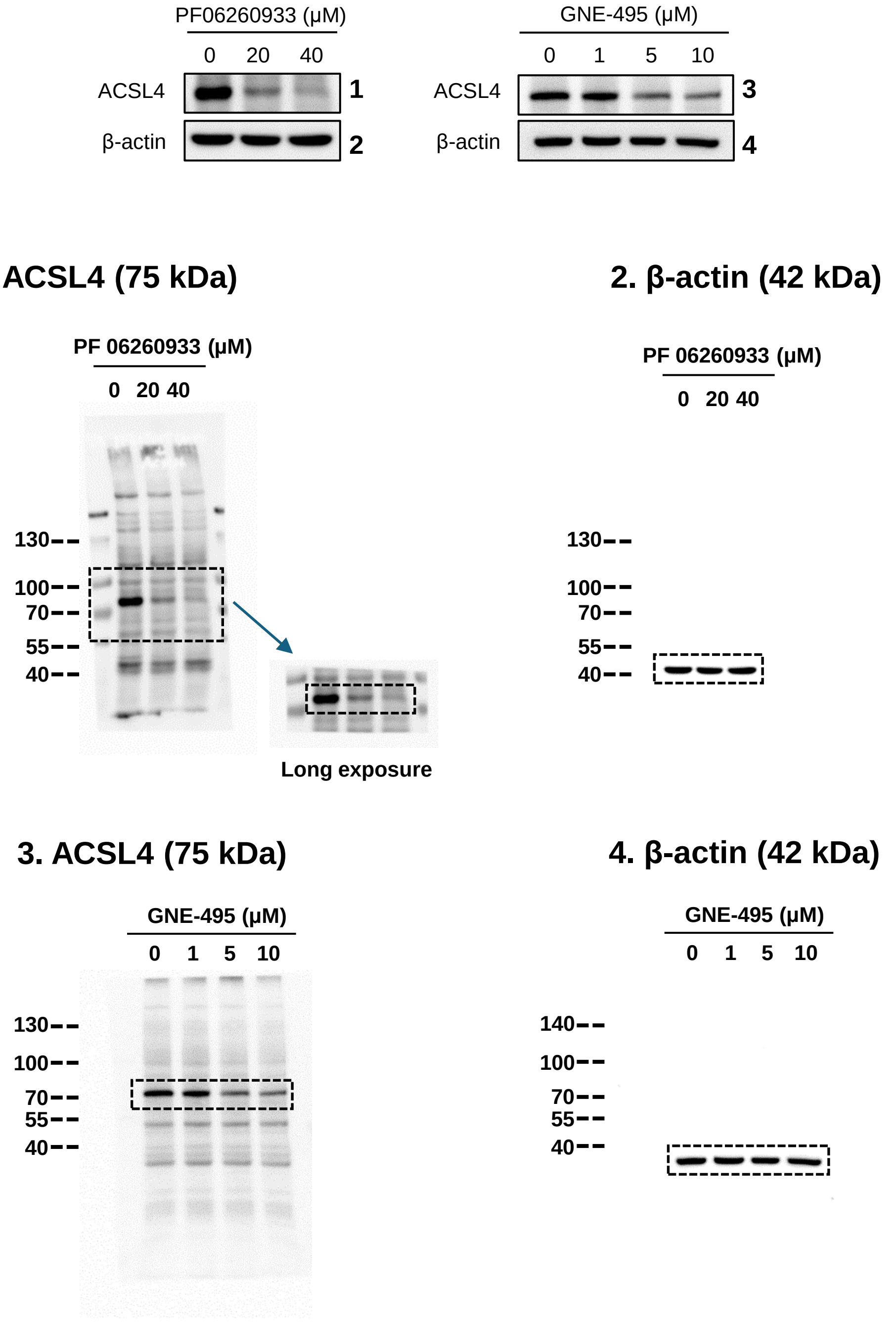

PF06260933 (μM)
0 20 40
ACSL4
β-actin
1
2
GNE-495 (μM)
0 1 5 10
ACSL4
β-actin
3
4
1. ACSL4 (75 kDa)
PF 06260933 (μM)
0 20 40
130
100
70
55
40
Long exposure
2. β-actin (42 kDa)
PF 06260933 (μM)
0 20 40
130
100
70
55
40
3. ACSL4 (75 kDa)
GNE-495 (μM)
0 1 5 10
130
100
70
55
40
4. β-actin (42 kDa)
GNE-495 (μM)
0 1 5 10
140
100
70
55
40

Figure 5C

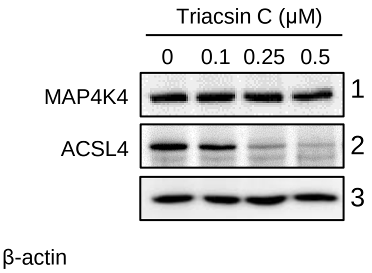

1. MAP4K4 (151 kDa)

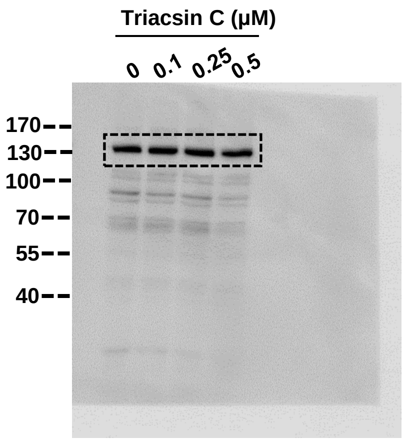

2. ACSL4 (75 kDa)

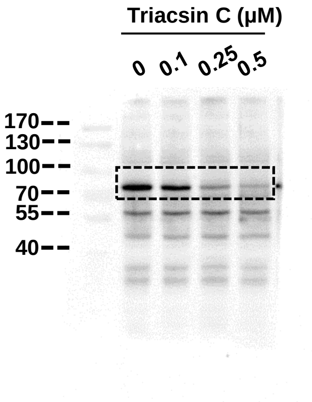

3. β-actin (42 kDa)

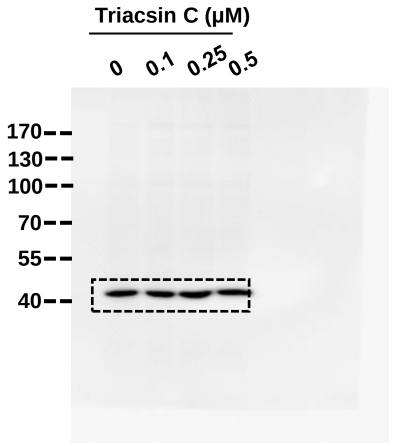

Figure 5E

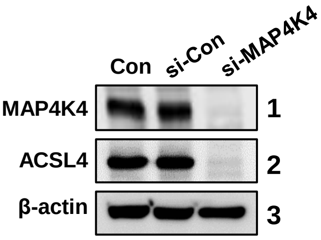

1. MAP4K4 (151 kDa)

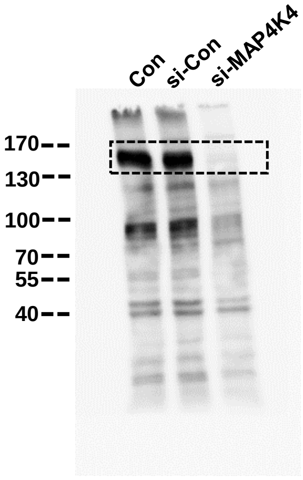

2. ACSL4 (75 kDa)

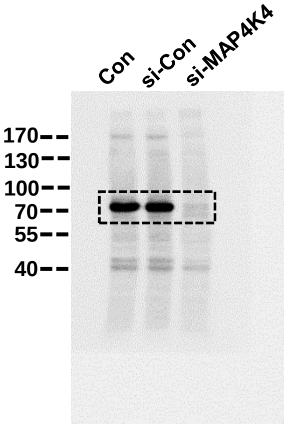

3. β-actin (42 kDa)

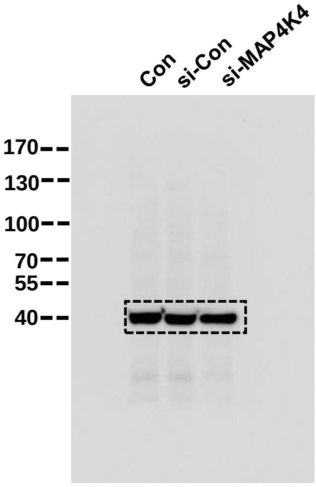

Figure 5F

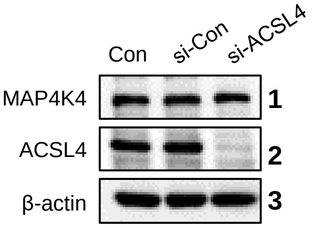

1. MAP4K4 (151 kDa)

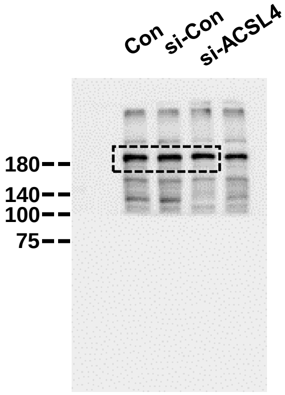

2. ACSL4 (75 kDa)

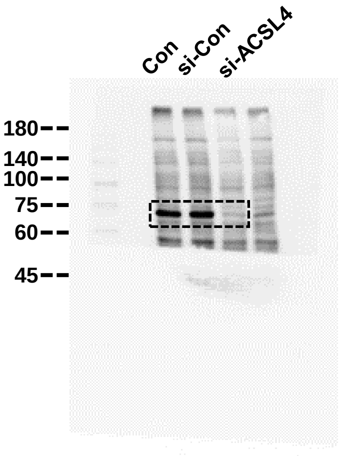

3. β-actin (42 kDa)

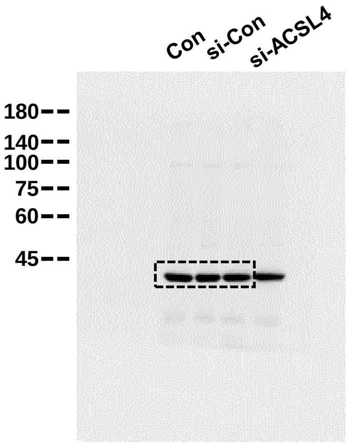

Supplement: Supplementary file 1 — Supplementary Figures. [file 41598_2024_57000_MOESM1_ESM.pdf]
